# Psychological factors affecting equine performance

**DOI:** 10.1186/1746-6148-8-180

**Published:** 2012-09-27

**Authors:** Sebastian D McBride, Daniel S Mills

**Affiliations:** 1 Royal Agricultural College, Cirencester, GL7 6JS, Gloucestershire; 2 Animal Behaviour, Cognition and Welfare Group, School of Life Science, University of Lincoln, Riseholme Park, LN2 2LG, Lincoln

**Keywords:** Psychology, Horse, Performance, Behaviour, Temperament, Mood, Emotion, Stress

## Abstract

For optimal individual performance within any equestrian discipline horses must be in peak physical condition and have the correct psychological state. This review discusses the psychological factors that affect the performance of the horse and, in turn, identifies areas within the competition horse industry where current behavioral research and established behavioral modification techniques could be applied to further enhance the performance of animals. In particular, the role of affective processes underpinning temperament, mood and emotional reaction in determining discipline-specific performance is discussed. A comparison is then made between the training and the competition environment and the review completes with a discussion on how behavioral modification techniques and general husbandry can be used advantageously from a performance perspective.

## Introduction

To attain optimal individual performance within any equestrian discipline, horses must be in peak physical fitness and have the correct psychological state. Professional riders acknowledge that these two factors are equally important and that without both, success is unlikely e.g. [1]. In addition, the relationship between a horse and its rider has been shown to be the most important factor when determining the risk of injury whilst riding [2]. However, despite its obvious importance for both performance success and human health, there is remarkably little research into any aspect of the psychology of equestrian performance. Psychological factors exist at three inter-related but separate levels: temperament, mood and emotional reaction [3]. Temperament exists as a relatively stable predisposition in adult life, which is shaped by both genotype and early experience [4], whilst mood describes a more temporary state of psychological functioning which helps to bias behavioral choices towards certain types of action in a predisposing environment [5]. For example, a negative mood, brought about by a series of aversive experiences in a given situation or over a particular period of time, may bias action towards escape and avoidance of novelty (and so serve to protect the organism from harm). Emotional reactions are the most tightly stimulus-bound affective states and the shortest lived temporally, thus, describing the more immediate response to the subjective evaluation of an event. If mood is negative then there is a higher probability of negative emotional reactions to a given situation [6]. Whilst there is a growing literature on temperament in horses (see [7] for review) there is still very little scientific work on the emotional reactions of horses and almost none on the assessment of moods. It is nonetheless important to appreciate that although it is difficult to study these phenomena, this does not mean that they are not important and certainly that they do not exist. Given the biological advantage served by these psychological constructs, this paper does not seek to present an argument for their existence but rather to evaluate their significance to equestrian performance. We start with a review of the role of temperament in performance, before considering the more proximate factors which can shape what a given horse may achieve at the time of a specific performance event. With this as a foundation, a comparison is then made between the training and the competition environment and the review completes with a discussion on how behavioral modification techniques and stress reduction through general husbandry may have the potential to enhance the performance of the horse.

### Temperament

Like physical traits, the psychological phenotype results from genotype-environment interaction and so has a measure of heritability. Physical and performance traits have regularly been used for breeding selection purposes with noticeable improvements in some countries. In the UK, for example, dressage performance increased from 1985 until 2001 (genetic standard deviation increases of 0.047 per annum) [8] and, since the introduction of performance tests in the mid-1980s in Sweden, using physical, performance and some temperament measures, the genetic progress has increased by 0.032 to 0.056 standard deviations per annum for dressage and show jumping respectively [9]. There has been some criticism, however, about the lack of objectivity within the temperament portion of these tests, primarily on the poor interpretation of behavioral data where multiple possible causal origins including environmental and rider effects are not being taken into consideration [10]. This criticism applies not only to professional performance tests but to equine temperament tests in general. Indeed, the absence of sound biological constructs or definitions of the various dimensions that make up temperament may be the primary reason why heritability values of temperament traits to date have been low. For example, Brockman and Bruns [11] reported a heritability of 0.26 for “temperament” in German Warmblood stallions, whilst the more precisely defined “jumping ability” in the same study had a much higher heritability of 0.62. It is, therefore, essential that measures are taken to objectively quantify strictly defined components of temperament, which should be based on sound biological theory, rather than arbitrary human choice.

A more precise definition of temperament and its components also helps define, in a more standardised way, the optimal genotype and thus aid the process of developing the optimal phenotype (from the perspective of discipline-specific competitive performance). What is crucial about the last statement is that optimal genotype is discipline specific; just as a Shetland will never win the Derby, so a horse of inappropriate temperament will generally never succeed within a certain discipline. It is therefore essential, not only to concisely define the biological basis of temperament, but also to identify very carefully which components (at the level of both specific behaviors and behavioral predispositions) are required to achieve success within a given discipline. For example, ‘flightiness’ may be generally advantageous to racehorse performance, but detrimental within the context of a dressage competition. Indeed, in a recent study by [12], although not the primary aim of the study, data did potentially highlight traits often sought within the different equestrian disciplines. The identification of these high performance discipline-specific behavioral traits can either be subjective (but in an informed away) as above (‘flightiness’ good for racehorse performance, detrimental within dressage competition) or can be achieved through a less subjective process of statistical correlation of well-defined traits with measures of performance.

In addition, greater precision in the definition of temperament traits also provides a better opportunity to select for that trait during the breeding-training process as opposed to, for example, constructs which are as complex and as multifaceted as 'eventing' resulting in low heritability values (0.20) [11]. It has also been suggested [13] that some studies of temperament in the horse tell us more about those evaluating the horses than the true biological basis of individual differences. For example Morris and colleagues [14] have suggested that horses share a similar personality structure to humans, but since they used a modified version of a human personality questionnaire describing subjective ratings, it seems somewhat inevitable that items would partition in a similar way. Nonetheless, appropriate questionnaire-based studies are valid and previous work in conjunction with behavioral studies has revealed at least three consistent temperament dimensions, which also accord with our current neurobiological understanding. One appears to relate to a sensitivity to aversion (often referred to as neuroticism in the psychological literature [15], flightiness in popular parlance e.g. [13] and is often assessed within behavioral tests by reactivity to a novel object e.g. [16], social isolation [17-20], and handling [19-21]. A second relates to a sensitivity to reward, (variously described as extraversion e.g. [14]; or in relation to exploratory behaviour in behavioral tests e.g. [22]. The third trait relates to sociability or gregariousness [23], again evident in the individual variation that occurs in response to social isolation [24]. The latter is obviously relevant when considering the background management of the athlete however we will focus this review on the former two traits relating more specifically to affective processing during training.

Temperament testing is often only practically useful if it is predictive of how an animal reacts to a range of situations over time. Whilst some studies have shown that individuals are consistent in their response between different tests [17,19,21], consistency over time within individuals has been more difficult to attain [18,25], although use of single behaviour measures (as opposed to combined forms [traits]) has been more successful in this respect [26-28]. This may be due to a) test-specific learning, a change in stimulus salience in relation to the test (a known artefact of repeat-testing especially in the case of “novel object tests”), b) added error through the additional computational step of multivariate statistical analysis, or c) general maturation effects of the animal. Nonetheless, it may still be possible to behaviorally profile immature horses and correlate these measures with performance success later in the animal’s life. Indeed, for Dutch show-jumping horses individual behavioral measures and combined forms of these data (traits) in response to ‘novel-object’, ‘handling’ and ‘learning’ tests were observed to be indicative of future jumping performance [25]. In particular, time taken to approach a novel object (open umbrella), ability to learn to avoid puffs of air in response to a bell sounding, and the combined behavioral trait of ‘sensitiveness’ (probably reflecting sensitivity to aversive stimuli) appeared to be particularly important in this respect. This particular study perhaps shows the value of objectively measuring behaviour in a range of well-defined tests and examining their biological commonality (e.g. sensitivity to aversion) and then correlating these with measures of performance, as opposed to measuring poorly defined traits which are preconceived to be relevant to a particular discipline (e.g. ‘flightiness’ for race horses).

Behavioral data of this nature also have the potential to be subjected to supervised statistical techniques e.g.discriminant function analysis [29,30]. Here, statistical software is informed about the performance success of animals (where failure is due to psychological rather than physical factors) to identify sources of variation within the behavioral screening data that are predictive of other animal’s potential performance success. Such a research project, at the national level, has the potential to save much time, effort and money, on horses that may have the physical but not the psychological aptitude for high-level competition success. However, one of the drawbacks of such an early screening strategy would be the risk of discarding athletically able young horses (on the results of an early temperament test), where potential behavioral problems during the middle to latter stages of the horse’s career could be resolved through behavioral modification techniques. The optimal approach in this respect would be to correlate early test results with performance of the animal after behavioral modification techniques had been applied.

#### Summary

In conclusion, out of the previously defined five personality dimensions considered to exist at various levels for different animal species [15], the horse reliably shows signs of two dimensions, neurotocism and extraversion, which relate to affective response, plus a third relating to the affective state associated with social needs. Although studies have consistently identified the presence of traits (within these dimensions) over time (in the same animals) as well as between animals, consistency of the value of the trait over time for individual animals has been demonstrated to a much lesser extent. This may be due to test-specific learning or general maturation processes changing the temperament of the animal over time. However, the predictive value of early temperament testing has been demonstrated with traits from both of the affective dimensions (as measured by novelty test, handling and learning) considered to be valuable in predicting jumping performance. Much more validation-type research measuring a full range of concisely defined early temperament traits for the purpose of subsequent correlation with discipline specific performance is required.

### Mood and emotional reaction

The importance of mood on performance is well recognised within equestrianism and scales such as the “Profile of Mood States” have been used to assess, through verbal report, various predispositions in the rider [31]. Obviously verbal report is not an option when assessing the horse, but work using heart rate variability (changes in inter-beat frequency) [32] suggests this may be a quantifiable alternative and a productive avenue for research for this species. For example, reduced heart rate variability (as revealed by analysis of the low and high frequency peaks in the power spectrum following Fourier transformation of the R-R data) suggests lower parasympathetic tone and thus higher arousal [33]. Thus, such measures in combination with behavioral data may help to determine, at the time of performance, whether high arousal relates to either a positive or a negative emotional state [34]. However, the relationship between arousal and performance is not a simple one. Excessive arousal, even if positive may be as detrimental to performance as under-arousal [35] and there is no optimal level that suits all. In human sports psychology, this individual requirement of arousal level has been termed the Individual Zone of Optimal Functioning (IZOF [36]). It is highly conceivable, therefore, that this same concept is also applicable to the horse [37] and, in this context, may be determined by correlating the aforementioned periodic sampling of heart rate with behaviour and performance of the individual animal. Whilst there may be little that can be done on the day of competition to alter mood, mood assessment before competition may be a very useful predictor of poor performance (as a result of both psychological and physical [e.g. subclinical onset of disease] factors) and could therefore be used to prevent injury or unnecessary unsuccessful competition. Mood assessment may also provide an additional and more objective means of determining how the horse is affected by different training and exercise regimes and thus how this impacts on performance. Such information might be of enormous competitive and welfare significance as it seems reasonable to suggest that, in the majority of cases, horses, like other athletes, will perform best when mood is positive. Recent developments in animal welfare science have offered up some robust experimental methods for assessing mood in non-human animals, although they have yet to be applied to the horse [38]. There may be a real advantage, therefore, for professional equestrians to integrate mood assessment techniques, both during training and at the point of competition, into their overall training strategy.

Emotional reactions differ from mood states in that they concern the immediate evaluation of the personal significance of a situation i.e. they are much more proximate in their temporal relationship with specific events and more tightly stimulus-bound compared to ‘background’ mood. Emotional reaction is mediated by the limbic aspect of the brain and primarily facilitates optimal preparation to a given situation on the basis of previous experience, but also serves a communicative function to conspecifics [39]. The latter allows emotional responses to be identified and measured behaviorally, although there can be disagreement over the exact emotion being expressed. The emotional reactions of horses to situations are evidently of enormous importance when it comes to their performance with over-reaction to environmental stimuli (reflecting a highly sensitive limbic arousal system) at the time of competition being the primary issue in this respect. However, the point at which advantageous emotional reaction becomes over-reaction and detrimental to performance varies between discipline. For example, during the highly constrained motor actions of dressage, it is important that the horse shows little or no additional motor response to non-rider environmental stimuli, whilst in show-jumping it is often considered that a higher level of emotional arousal can be tolerated. For high racing performance on the other hand, high emotional arousal is considered a pre-requisite but again not to the point where it becomes deleterious e.g. the horse not loading into the starting gate or over-energy expenditure preventing the horse from going to distance or responding to the rider in the final furlong. Given that emotional responses are partly dependent on previous experience, the method of training used is, therefore, critically important when trying to build optimal individual performance. The majority of training is based on the use of aversive stimuli in the form of either punishment to discourage undesirable behaviour or negative reinforcement to encourage appropriate behavior [40]. For the latter, it is the removal of the aversive stimulus which provides the reinforcement for the correct behavior and which, with consistency, leads to early anticipation and avoidance of the training aid so the animal becomes responsive to the most subtle cue from the rider. Timing is therefore critical and poor timing may lead to the learning of unanticipated and inappropriate responses [40]. Although employed to a much lesser extent, training can also be achieved through positive reinforcement. Because responses are associated with reward acquisition, they are much more variable as, evolutionarily speaking, it pays an animal to explore the limits of what is required to obtain a reward so it can maximise efficiency through minimal effort [41]. However, the key issue with positive reinforcement (and where it contrasts most with negative reinforcement and punishment) is that emotional responses to the training situation are often entirely positive rather than largely or wholly negative [42,43]. This may be extremely important in shaping the horse’s perception of being ridden and the relationship which develops between the horse and rider, as a result (which may be particularly important when the rider and the trainer are the same person).

Somewhat surprisingly, given that in many disciplines, success depends on an optimal partnership rather than excellent individuals [1], very little is known about the effect of the rider’s emotional state on that of the horses [37]. Horses are known to react differently when stroked by someone with a negative attitude to them compared to someone with a more positive attitude [44], and so they may detect changes in rider behavior due to such things as competition anxiety. More recent findings also tentatively suggest that both the rider's and horse's personality affect the level of cooperation between the two [45] thus supporting the common anecdote that some horses suit some riders. This is an area of research that again requires much more exploration, potentially through assessing the personality factors within the horse-rider dyad and ultimately correlating this with measures of performance.

#### Summary

In conclusion, both mood and emotional state are crucial in determining how the horse perceives and reacts to its environment and thus how it will perform within a training and competition environment. Positive mood is essential for all disciplines but the optimal emotional state leading to optimal emotional arousal can vary between disciplines and between horses, as in the case with humans (IZOF). Over-reaction as the result of high emotional arousal is detrimental to performance and is heavily influenced by prior training techniques and also the emotional state of the rider. More extensive research is required within both of these areas.

### Training versus competition environment

Many horses may fail in competition because of the difference between the training and competition environments and thus the lack of training to generate appropriate emotional (and thus behavioral) responses to the latter. The purpose of these sections is to again highlight practical approaches and potential areas of scientific study that may be of benefit in this regard from performance perspective.

#### Training environment

Training has been defined as ‘suppressing undesirable natural responses, exploiting desirable natural behaviour and instilling novel behaviour by the deliberate or accidental application of learning theory’ [46]. For the performance horse, training obviously also involves conditioning of the cardio-vascular and musculoskeletal systems, and the two (psychological and physical) are normally inextricably intertwined. Two additional psychological factors that are frequently referred to in relation to learning and training ability are intelligence [47] and motivation [48,49]. Just as with the emotional constructs discussed above, these concepts are often poorly defined, in fact, it has been argued that the use of the term “intelligence” to infer a continuous scale of ability in relation to learning is probably inappropriate as horses that perform well in one type of learning task may not necessarily perform well in another (see [47] for review). This is not perhaps surprising because a) tasks are predominantly based on operant learning; the animal performs a task in order to either avoid negative reinforcement (e.g.discomfort or pain) or attain positive reinforcement (e.g. food [primary reinforcer] or verbal praise/ clicker [secondary reinforcer]), b) animals differ in their sensitivity to reward and aversive stimuli (as previously discussed) and c) different balances of reward and aversive stimuli are applied within each learning task. Indeed, a recent study by Lansade and Simon [50] clearly demonstrated a correlation between aversion sensitivity and ability to learn via a negative but not a positive reinforcement paradigm. Given that different disciplines or training regimes within discipline also require different balances of reward versus aversive stimuli, it may therefore be more appropriate to discuss learning ability of individual horses in the context of discipline-specific tasks, as listed, for example, in Table 1). However, it should also be noted that most tasks can be achieved using combinations of both positive and negative reinforcement, therefore, from a practical perspective, it is perhaps more useful to ascertain individual sensitivity to reward and aversive stimuli as a way of identifying the most effective training strategy. As previously discussed, behavioral methods of assessment (positive and negative reinforcement learning trials) already exist (see [47] review), but much more research is required to create more practical tests that could easily be applied pre-training within a short period of time to help determine the optimal training strategy (negative versus positive reinforcement) for the individual performance horse. A potential starting point for this work could be the simplification of experimental psychology studies that have previously quantified reinforcer sensitivities in the horse [51] and other species [52,53].

**Table 1:** A sample list of tasks (and their relative level of complexity) associated with racing, show-jumping and dressage training

**Discipline**	**Task**	**Type of learning process**	**Task complexity**
Racing (all types)	Loading into the starting gate.	Habituation and operant conditioning (negative reinforcement)	Low
Racing (all types)	Increased or decreased speed of gallop in response to aids (bridle, leg and crop)	Operant conditioning (negative reinforcement)	Low
Racing (National Hunt)	Jumping	Motor-coordination	None
Show jumping	Increased or decreased collection in response to aids (bridle, leg and crop)	Operant conditioning (positive and negative reinforcement)	Medium
Show jumping	Jumping	Motor-coordination and operant conditioning (positive and negative reinforcement)	Medium
Dressage	Specific dressage moves ranging in complexity from collection to piaffe	Operant conditioning (positive and negative reinforcement)	Medium-high

The second factor highlighted as being important to training was motivation. It is considered that motivation, for a range of species, can originate both cognitively (within cortical regions of the brain) and emotionally (from sub-cortical regions) involving focus on specific goals associated with either the attainment of something the animal considers to be desirable (e.g. food) or, the avoidance of that which is aversive (e.g. pressure, pain) [54,55]. These are the same fundamental end points that drive learning processes and thus, motivation and the attainment of goals are intertwined within a cognitive learning process. Although it has been argued that for horses, athletic activity itself may be rewarding and thus horses may be intrinsically motivated to work or exercise [56], the majority of training requires additional incentive. Normally this originates from avoidance of pressure or pain (negative reinforcement) and occasionally involves the use of reward (e.g. food) or associated secondary reward (e.g. verbal praise or clicker) in the form of positive reinforcement. The level of motivation for the attainment of goals in horses varies dramatically between individuals and again may reflect individual sensitivity to reward and aversive stimuli [22]. Thus, from a performance perspective it is perhaps again more appropriate to talk, not about learning ability, but rather motivation to learn based on the animal’s basal motivation to avoid negative and attain positive reinforcers. For highly complex tasks (for example in dressage), learning ability for that task (‘intelligence’) will have greater importance [57]. However, high level of motivation will still be paramount for successful task completion. It follows, therefore, that one of the most important factors at the outset of training is to ensure that the individual animal is sufficiently motivated to perform. Interestingly, in human sports performance psychology, this motivation either to succeed or to avoid failure is so enhanced in some individuals that it often develops clinically as obsessive-compulsive characteristics, referred to as ‘perfectionism’ [58-60]. To the extent that it has raised the question in the human literature as to whether such human personality characteristics are now a prerequisite for sport success. It may also be that such high motivation characteristics are also a pre-requisite for the modern equine equivalent. Interestingly, the neurochemical pathway considered to be intrinsic to motivational processes demonstrates significant variation in activity between individual horses [61]. Individuals with higher activity (and thus potentially greater motivation for goal-directed behaviors) also differ behaviorally in that they are a) more prone to stereotypic behavior and b) persist more within a positive reinforcement operant task (continue pressing the food dispensing button) when the reward that they are working for is taken away [62]. From a practical perspective, although these studies may suggest that the level of basal goal-directed motivation (at least in the context of positive reinforcement) could actually be tested for, it also demonstrates that such a selection strategy could lead to a greater incidence of stereotypic behaviour within that equine sub-population. This raises the moral dilemma that often exists within animal production/performance systems that selection of one trait (in this instance high motivation) for the purpose of enhanced production/performance may be detrimental to the animal from a welfare perspective.

Motivation to learn is also heavily affected by the learning environment, in particular the duration of the training session and how frequently those sessions occur on a daily or weekly basis. For example, horses in a negative reinforcement situation took fewer training sessions to learn a task when those sessions took place once instead of two or seven times a week [63] and the number of trials within a session has also been demonstrated to be important in the context of optimal learning [64]. Again the level of motivation to perform will be determined by the type of learning taking place (e.g. negative versus positive reinforcement) and the complexity of the task. In this respect, much more research is needed to establish optimal training schedules for the specific tasks listed in Table 1.

#### Competition environment

The competition environment is considerably different to that of the training one in several respects; 1) the presence of other horses (except for racing), 2) additional visual and aural stimuli, and 3) conditioned stimuli that signal a competitive event. Each of these factors will elicit an emotional response in the animal that will affect the motivation of the horse towards the set task on the day of competition. For many horses, these factors enhance arousal, increase ‘excitability’ and lead to a general increase in locomotory behaviour. The latter often have to be restricted (given the competition environment) which can result in an acute stress response in the horse. However, for some disciplines, the physiological consequences of acute stress, i.e. energy mobilisation and increased cardio-vascular activity, can be beneficial. For example, the presentation of novel stimuli (known to induce an acute physiological stress response) pre-race has been found to enhance running performance in Thoroughbreds [65]. Overly aroused horses, however, can become difficult to handle, expend too much energy before the competition or become distracted in a way that detracts from performance as they move out of their previously described ‘Individual Zone of Optimal Functioning’ [37].

Factors associated with the competition environment can also result in enhanced motivation to perform other non-competition behaviors. This is normally a consequence of either 1) the horse being fearful of novel visual and aural stimuli associated with the competition environment and/or 2) previous negative experiences (e.g. pain) associated with the competition environment. Both result in motivation and behaviour focussed on exit from that environment (flight response). Behaviorally, it can be difficult to differentiate between the aforementioned ‘excitability’ (as a result of restricted motivation to perform) and enhanced locomotory response to novel stimuli or fear. It is important, however, to make this distinction in the emotional response of the animal if behavioral modification techniques are to be applied (discussed in the next section).

#### Summary

In summary, for the purposes of successful competition regardless of the discipline, it is important that the horse is highly motivated to perform the specific athletic activity at the outset of training and competition. The animal should also respond in a highly motivated way to positive and negative reinforcement techniques during training to facilitate modification of athletic activity. However, it is important to identify the individual sensitivity to these reinforcers in advance to ensure optimal training strategy and much more research needs to be done in this respect to establish practical and reliable tests for the performance horse owner. A starting point for this work could potentially be based on experimental psychology studies that have quantified reinforcer sensitivities in the horse [51] and other species [52,53].

The performance horse also needs to be motivated at the time of competition, but not to the extent that any restriction of that motivated behaviour has a negative effect on the animal’s physiological or psychological state. Highly motivated horses, however, can be exposed to behavioral modification techniques in order to attenuate specific unwanted behaviors but the animal must be capable of responding to these techniques in a positive way. Modification techniques can also be applied to highly reactive horses that are responding to novel stimuli or previous negative experiences, but again those individuals need to be responsive to those techniques.

### Behavioral modification

In light of the previous discussion, the main areas where behavioral modification could potentially be applied to enhance performance are:

1) Basic positive and negative reinforcement techniques to aid motivation towards the correct athletic behaviour;

2) Counter-conditioning and systematic desensitisation to attenuate overly reactive behaviour in anticipation of the competitive event;

3) Counter-conditioning and systematic desensitisation to attenuate overly reactive behaviour to novel stimuli associated with the competitive event;

4) Counter-conditioning and systematic desensitisation to attenuate motivated behaviors in response to stimuli associated with the onset of a competitive event that are a result of previous negative experiences linked to those stimuli.

As previously discussed, identifying individual sensitivity to reward and aversive stimuli is considered crucial in determining the individual optimal training strategy from a positive versus negative reinforcement perspective. However, two other issues surrounding the different reinforcement techniques need to be taken into consideration when devising a training approach. Firstly, long-term inappropriate application of negative reinforcement schedules may result in a chronic stress situation for the animal, potentially leading to reduced health [66], high reactivity to acute stressors [67], or, for some individuals ‘learned helplessness’ (behavioral depression) [68]. Secondly, positive reinforcement training methods may have limitations in the amount of work the animal will perform for the reward [69] resulting in greater likelihood of refusal to perform psychologically or physically demanding tasks. Given that all companion animal species have evolved in environments where both positive and negative reinforcement occurs, it is generally considered that a combination of both schedules [70] are the most efficient in terms of terms of training and are less likely to affect other aspects of the horse either behaviorally or physiologically. Testing for reward versus aversion sensitivity within the individual horse, therefore, will again help determine the optimal balance of reinforcement schedules (negative versus positive) to be used rather than an exclusion of one over the other.

Habituation, as a method to reduce reactive behaviour to novel stimuli also has its limitations in that some individual horses do not attenuate their behavioral response to repeated exposure of the stimuli, although this can be managed through the use of systematic desensitisation [71]. Anecdotally, this difficulty has been recognised for some time, however, it has also been recently demonstrated within a controlled experimental situation that individual horses appear to adopt either one out of two adaptive strategies to repeated exposure of a novel stimulus, either habituation (reduced physiological and behavioral response) or sensitisation (increased physiological and behavioral response) (McBride, unpublished data). In this context, it is important to identify the phenotype in order to apply the correct modification techniques that will yield productive results. In the absence of the necessary stimulus control for systematic desensitisation, counter-conditioning is an appropriate alternative behavioral modification strategy for the latter group of horses where animals are taught to perform a behavior which is incompatible with the unwanted behavior (e.g. standing to replace locomotory behavior), normally with the use of a positive reinforcer [40].

Previous negative experiences linked to stimuli associated with the onset of a competitive event not only generates a range of motivated behaviors that have the primary aim of removing the animal from that situation (for example [72]), but it can affect motor function in a way that is normally interpreted as reduced confidence. In human sports psychology, confidence along with ‘mental toughness’ and ‘motivation’ is one of the primary factors that determines competitive athletic performance [73]. As stated, confidence is based primarily on prior experience, where that experience has not been persistently negative for the animal. In this respect, the rate of training and physical demand within an equestrian discipline is extremely important so that tasks set do not become aversive by being outside the horse’s physical capability, either in terms of strength or motor co-ordination. Incorrect rate of training can also increase the chances of injury to the animal which will again be perceived as a negative event affecting subsequent performance. As previously stated, negative experiences also induce an anticipatory stress response on re-presentation of the same situation. Stress affects motor control to reduce motor co-ordination [58,74,75], thus, the animal is more likely to perform motor error potentially resulting in further injury. A perpetual cycle of anticipatory stress followed by injury can be categorised, in the context of conventional learning theory, as ‘punishment’ and will have drastic effects on the performance of the animal. Conventional methods to counter this condition are to reduce the physical demand of the exercise to allow the animal to perform the task without injury, followed by increased demand built up slowly over time thus restoring the animal’s ‘confidence’. It should also be noted, however, that in humans, athletic confidence has also been directly related to the individual’s general personality [76] regardless of experience. This area again needs much more research with regard to the performance horse.

### Non-competition and non-training stressors

It should be noted that stress affecting performance does not necessarily have to originate from sources associated with training or the competitive event. Performance horses are exposed to a range of stressors most of which relate to the husbandry of the animal (often dictated by the training regime) and are stressful because they affect the behavioral needs of the horse as a species [77]. Behavioral needs are species-specific highly motivated behaviors that are performed irrespective of their functional consequence [78]. More often than not they do have a functional role and human-intervention to pre-provide the consequence of the behavior may not reduce the motivation for its performance i.e. there appears to be some physiological ‘need’ to perform the behavior regardless of what that behaviour brings to the animal. Foraging is a primary example in this respect [79]. For many herbivores, forage is naturally available ad libitum with up to 70% of the day spent eating [80]. For horses, the reduction of eating time to two meals per day can meet the nutritional requirements of the animal, but may not necessarily meet the animal’s behavioral need to forage [81-83]. The problem with restriction of behavioral needs from a competition perspective is that it induces a chronic stress response in the animal [69,84] which will subsequently prevent optimal individual performance.

Two other considered behavioral needs of the horse as a gregarious ungulate, are social interaction and locomotory behaviour where again the restriction of these behaviors are considered to reduce the animal’s welfare [56]. Thus, optimal environmental conditions, to maintain the horse at a high performance level, are those that facilitate the behavioral needs of the species, thus reducing the risk of a chronic stress situation.

Other potential sources of stress for the competition horse include transportation [85] and over-exercise. The latter is well recognised in the field of human sports science and is related to the condition of ‘burn-out’ [85], resulting in reduced motivation towards training and athletic competition [86,87]. The primary causal factor of ‘burnout’ appears to relate to insufficient positive feedback (reward) for work performed and again there appears to be individual genetic susceptibility in this respect [88]. This condition is again anecdotally recognised in horses but no research has been carried out to ascertain what level of exercise and type of training brings it about within the different equestrian disciplines and whether it is possible to predict individual predisposition in this respect.

## Conclusions

The increased competitiveness and performance level of sport now requires that individuals and teams must give over a substantial amount of time to their respective disciplines. However, even when an optimal training infrastructure has been attained, successful competition is now only achieved through the additional integration and application of sports/exercise science and technology. Although it is considered that this is the antithesis of sporting ethos [89], it is without doubt a considered pre-requisite for international sporting success [90].

This review has identified areas within the current performance horse industry where known behavioral research and behavioral modification techniques could be applied to enhance further the performance of those animals. These include:

1. current research on equine behavioral needs to ensure optimal environmental conditions;

2. the application of behavioral modification techniques to:

a. sufficiently motivate the animal to perform the correct athletic behaviour;

b. attenuate overly reactive behaviour in anticipation of the competitive event;

c. attenuate emotionally reactive behaviour to novel stimuli associated with the competitive event;

d. attenuate motivated behaviors in response to stimuli associated with the onset of a competitive event that are a result of previous negative experiences linked to those stimuli.

This review has also identified areas of further research that could potentially enhance the performance horse industry. These include:

1) the development of a behavioral screening tool to identify young horses that do not have the correct temperament in order to proceed to the top level of competition within a given equestrian discipline;

2) the integration of methods aimed at assessing the emotional state of the horse during training and competition in order to ensure that the horse is in an appropriate psychological state for competition.

3) the identification of optimal training regimes in terms of applying positive and/or negative reinforcement schedules and also in terms of training duration and training interval with the primary aim of avoiding the equine equivalent of psychological ‘burn-out’.

Finally greater work is required on the rider-horse partnership in order to identify the constituents of a winning team within a given discipline.

## Competing interests

The authors declare that they have no competing interests.

## Authors’ contributions

Authors SD McBride and DS Mills contributed equally to the writing and preparation of this manuscript. Both authors read and approved the final manuscript.
